# Bead mediated separation of microparticles in droplets

**DOI:** 10.1371/journal.pone.0173479

**Published:** 2017-03-10

**Authors:** Sida Wang, Ki-Joo Sung, Xiaoxia Nina Lin, Mark A. Burns

**Affiliations:** 1 Department of Chemical Engineering, University of Michigan–Ann Arbor, Ann Arbor, MI, United States of America; 2 Department of Chemical Engineering, Massachusetts Institute of Technology, Cambridge, MA, United States of America; 3 Department of Biomedical Engineering, University of Michigan–Ann Arbor, Ann Arbor, MI, United States of America; Texas A&M University College Station, UNITED STATES

## Abstract

Exchange of components such as particles and cells in droplets is important and highly desired in droplet microfluidic assays, and many current technologies use electrical or magnetic fields to accomplish this process. Bead-based microfluidic techniques offer an alternative approach that uses the bead’s solid surface to immobilize targets like particles or biological material. In this paper, we demonstrate a bead-based technique for exchanging droplet content by separating fluorescent microparticles in a microfluidic device. The device uses posts to filter surface-functionalized beads from a droplet and re-capture the filtered beads in a new droplet. With post spacing of 7 μm, beads above 10 μm had 100% capture efficiency. We demonstrate the efficacy of this system using targeted particles that bind onto the functionalized beads and are, therefore, transferred from one solution to another in the device. Binding capacity tests performed in the bulk phase showed an average binding capacity of 5 particles to each bead. The microfluidic device successfully separated the targeted particles from the non-targeted particles with up to 98% purity and 100% yield.

## Introduction

Bead-based microfluidic technology has been used extensively in research in recent years for applications such as protein analysis [1,2], bacterial detection systems [3,4], and other biological applications [5,6]. Bead-based microfluidic systems hold many advantages over conventional techniques including low reagent consumption, faster reaction times and high sensitivity [7]. These systems pair well with droplet microfluidics as droplet-based devices offer the same advantages and can be used for the same types of applications [8]. Previous papers have demonstrated the use of droplet microfluidics for PCR [9,10], cell growth, and cell sorting [11–13]. In these applications, droplets must remain intact and distinct from one another for long periods of time. To keep droplets separated requires the use of surfactants to decrease surface energy and prevent droplet merging [14]. However, difficulties arise when droplets need to be manipulated for further downstream analysis. In particular, techniques such as merging [15,16] and separation of droplet content become challenging since these actions require the manipulation of the droplet interface. Additional challenges to designing a viable method for these techniques include the need to maintain separate and distinct droplets after performing the manipulation.

Separating components in a droplet or moving a component from one droplet to another is a complex technique that is important in assays such as enzyme-linked immunosorbent assay (ELISA) and fluorescence in-situ hybridization (FISH). With this technique, the goal is to maintain a component or target in the droplet while removing the rest of the unwanted solute and solution, and the technique should ultimately be able to be achieved in a high-throughput manner. State-of-the-art work in the segregation of droplet content focuses primarily on two areas: electrowetting on diodes (EWOD) and magnetic particles to separate droplet content. EWOD manipulates the effective droplet surface tension and wetting properties by an electric field [17,18]. Various techniques such as merging, splitting and separation have been demonstrated in previous works using this concept [19–21]. Techniques using magnetic beads bind the target and separate them from the rest of droplet by a magnetic field generated on-chip. Works by Brouzes *et al*. and others focus on separating content by pulling magnetic beads in one direction while the rest of the droplet is split [22–25]. Another paper by Kim *et al*. demonstrates the separation by pulling the magnetic beads through a series of aqueous and oil phases separated by posts [26]. All of the above-described systems require fabrication or use of an electric or magnetic system on-chip. Some other works have demonstrated separation or enrichment using acoustic waves [27,28] or flow fields [29,30].

In this work, we fabricated a PDMS particle separation device that uses surface functionalized polystyrene beads to capture target particles capable of separating surfactant-stabilized droplets. Beads are trapped in a row of posts, and a new droplet is generated on-chip to re-encapsulate the trapped beads thus retaining the target particles. We determined optimal operating parameters and demonstrated successful capturing of the particles. Then we tested the capability of the bead’s binding to the target particles. Finally, using our device and surface functionalized polystyrene beads, we demonstrated the exchange of droplet content during which targeted particles were retained whereas non-targeted particles were removed.

## Materials and methods

### Materials

Beads for testing capture efficiency at the posts were 1.99 μm (Spherotech), 4.16 μm (Spherotech), 6 μm (Interfacial Dynamics Corporation), 7.32 μm (Bang Laboratories, Inc), 8.62 μm (Polysciences, Inc), 10.2 μm (Spherotech), 11.3 μm (Spherotech), and 16.2 μm (Spherotech) in average diameter.

In droplets experiments, the continuous oil phase is fluorocarbon oil (HFE-7500, 3M) containing 2% perfluoropolyether-polyethyleneglycol surfactant (RAN Biotechnologies). It is made with 10% stock solution through sonication and dilution into a 2% solution. The dispersed aqueous phase consists of 1X PBS (Fisher Scientific) with 0.05% Tween-20 (Sigma Aldrich) in the original and recaptured droplets. 14–17.9 μm diameter polystyrene beads functionalized with streptavidin (Spherotech) were used as the capturing beads. Pink fluorescent polystyrene particles with diameter range of 1.7–2.2 μm (Spherotech) were used as the target particle. Green fluorescent latex beads (Polysciences, Inc.) with diameter range of 1.7–2.2 μm were used as the non-target particle. Polydimethylsiloxane (PDMS) (Sylgard 184 Silicone Elastomer, Dow Corning), silicon wafers (Silicon Valley Microelectronics), and SU-8 (Microchem) were used for device fabrication.

### Fabrication of PDMS device

Photomasks were designed on L-Edit and made in the Lurie NanoFabrication Center at the University of Michigan. The SU-8 mold was made by negative etching on a silicon wafer. The silicon wafer was spin-coated with SU-8 2035 at a thickness of 50 μm. The wafer was pre-baked at 65° C and then at 95° C. The wafer was then exposed and a post-exposure bake was performed at 95° C. After baking, the wafer is silanized with (tridecafluoro-1,1,2,2,-tetrahydrooctyl)-1-trichlorosilane using a desiccator. PDMS is poured on top of the SU-8 mold, vacuumed to remove air bubbles and heated to solidify the polymer. The devices are cut, punched with holes to create openings for the channels, and bonded on glass slides using plasma-activated bonding using a corona discharge wand.

### Particle binding to beads

Streptavidin beads were mixed with pink fluorescent biotinylated particles, green fluorescent non-biotinylated particles, and a combination of both at various ratios in 100 μL of 1X PBS with 0.05% Tween-20. Beads and particles were incubated overnight in room temperature or on a Fisher Scientific vortexer mixer set to shake at a setting of 1 (300 rpm) in a microcentrifuge tube. Beads and particles were centrifuged at 5,000 rpm on a centrifuge for one minute. Resuspension was performed using a vortexer set at max rotational speed (3200 rpm). Beads are separated from non-bound particles by a 5 μm filter. The beads are washed off the filter and resuspended in PBS. All counting of particles bound was done via microscopy by fluorescence to identify targeted and non-targeted particles.

Beads and particles were counted using a hemocytometer to determine their concentrations. They were then diluted to generate one streptavidin-functionalized bead and 25 mixed targeted and non-targeted particles per droplet. Droplets were generated on a flow-focusing PDMS droplet generation device. Droplets are collected using an Eppendorf tube that has tubing and a syringe connected to it. Droplets are incubated overnight at room temperature.

### Droplet generation and device operation

The device is operated using pressurized air to flow the droplets and solutions in the channels. Droplets are introduced in the droplet inlet/collection channel by applying pressure to the syringe connection. There are three inlets connected to the pressure source by a syringe tip, tubing and syringe. The pressure is controlled by a voltage box, which regulates the pressure range between 0–5 psig. The voltage is regulated using a LabVIEW program. Droplets previously generated are introduced by applying a pressure of 0.028 to the droplet inlet channel. A pressure of 0.016 psig is applied to the spacing oil channel and aqueous channel while flowing the droplet toward the post region. Once the bead has been captured, the pressure in the aqueous phase is increased to 0.020 psig to generate a plug. The pressure is decreased to 0.016 psig once the plug has been generated. The pressure is turned off once the plug has re-encapsulated the bead and the plug becomes the desired droplet size. Droplets that contain the retained beads and particles are taken out of the device through the droplet inlet. All counting of particles bound was done via microscopy by fluorescence to identify targeted and non-targeted particles.

## Results and discussion

### Device operation and characterization

**Fig 1:**
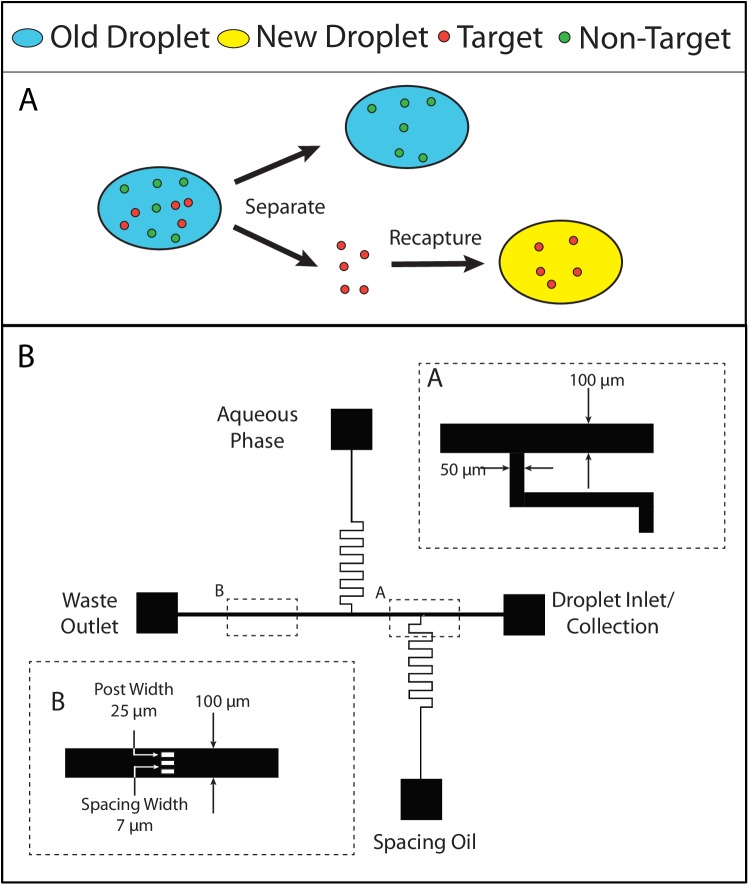
Targeted separation concept and device schematic. (A) Overall operation of separating a target in a droplet and recapturing the target in a new droplet. (B) Schematic of the device used for the droplet separation device. Zoomed in image A show the dimensions of the main and side channels. Zoomed in image B shows dimensions of the posts for bead capturing.

**Fig 2:**
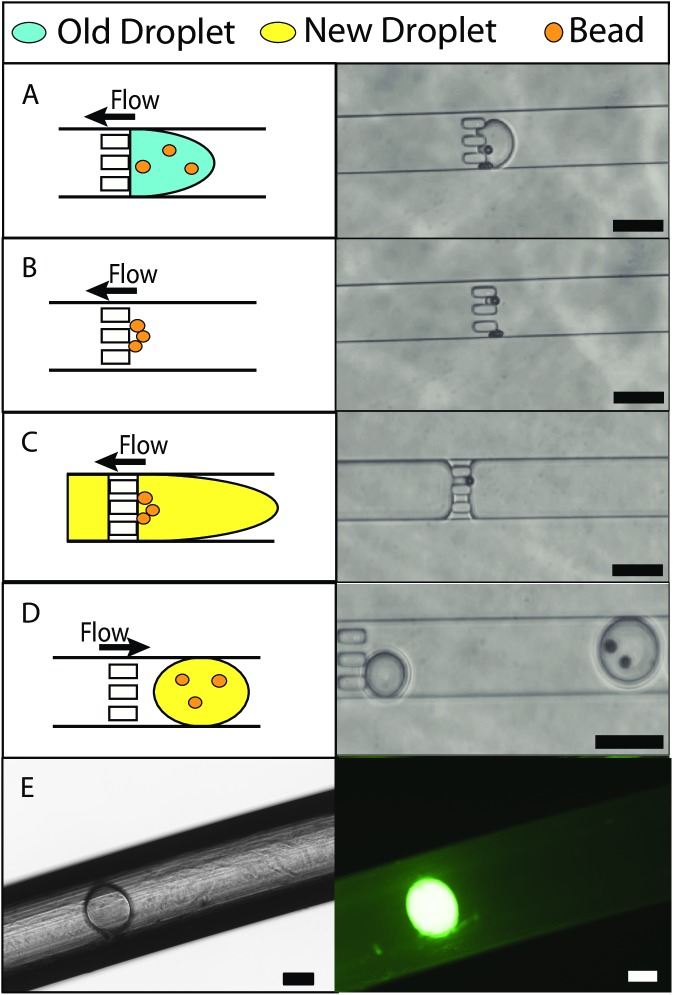
Bead separation schematic, images, and validation of droplet extraction. (A-B) Schematic and images shows process during bead capture. See S1 File. (C-D) Schematic and images shows process of bead re-encapsulation. Note for image D which shows two beads that the beads were overlapped in the previous image. See S2 File. S2 Fig shows an image with bead re-captured with a target bound onto the bead. (E) Image of a fluorescent droplet taken out of the device and captured in a capillary tube. Scale bars are 100 μm.

Separation of a target in droplets requires several steps: separation of the target from the droplet and re-encapsulation of the target in a new droplet (Fig 1A). The microfluidic device has several features that are used to capture the beads, generate new droplets and recapture the beads. The generated droplets contain beads and target microparticles, which bind to the surface of the bead via streptavidin-biotin. Non-targeted microparticles with no surface functionalization were used for the negative control. Droplets can be introduced from the inlet channel and become trapped at the posts located downstream of the main channel (Fig 1B). New droplets are generated in the aqueous channel at the T-junction with the main channel. The beads that are trapped can be recaptured with the newly generated droplets and removed from the device through the droplet inlet channel. The device operation process is summarized in Fig 2A–2D. A droplet containing fluorescein and no fluorescein are removed from the device and stored in a capillary tube is shown Fig 2E and 2F respectively. The images demonstrate the ability to transport and store an intact droplet out of the device for future analysis.

The capture region of the device was designed with 6 μm gaps to optimize both bead capture and fabrication. However, the actual post spacing was measured to be between 7–8 μm due to deformation of the device during fabrication. The device is designed so that only a single droplet is moved to the posts, preventing multiple droplets from entering this region and/or merging into larger droplets. Once a single droplet reaches the posts, the resistance in the channel increases due to the surface tension of the droplet and its interaction with the posts. The increase in resistance prevents subsequent droplets in the inlet reservoir from entering the channel. This phenomenon allows for the capturing and recapturing of beads from a single droplet. To ensure that subsequent droplets do not enter the channel once a droplet has completely passed the posts, the pressure applied to the inlet is decreased. Note that, because the presence of the posts increases the fluidic resistance, pressure at the aqueous and spacing oil reservoirs also had to be increased slightly. In addition, 90 degree bends in the oil and aqueous channels increased resistance in those channels.

Accurate pressure settings are crucial for successful device operation. Laplace pressure required to push the droplet through the inlet was determined by the Young-Laplace equation:

$$\Delta P=\gamma \left(\frac{1}{R_{1}}+\frac{1}{R_{2}}\right)\;\cos\;\theta$$ (1)

where ΔP is the Laplace pressure, γ is the surface tension of the oil with surfactant, R_1_ and R_2_ are the principle radii of curvature and θ is the contact angle. The droplet was assumed to have a spherical shape for the radii of curvature. The surface tension of the oil with surfactant was determined from a paper by Brousseau et al. [31] that utilizes the same oil and similar surfactant. Experimentally, the contact angle was observed to be approximately 60–70° before the droplet enters the post region. The Laplace pressure was calculated at least 0.210 psig. The experimental Laplace pressure needed to push the droplet through the post was 0.044 psig indicating a higher contact angle (approximately 86° in the post region). This difference could be a result of many factors including differences in shape at the post edges and dimensions of the post channels. Note that Pressures greater than 0.044 psig can result in multiple droplets entering the post region before recapturing and Pressure lower resulted in insufficient pressure to drive the droplets into the device inlet and push the droplet interface through the posts. New droplets were generated by calibration of pressures in the channels to ensure that the bead remains trapped on the posts while a new droplet is being generated. When the plug is being pushed through the posts, the final size can be manipulated by stopping the flow when the plug becomes the droplet of desired size. The total time of operation for the device is 3–4 minutes per droplet.

**Fig 3:**
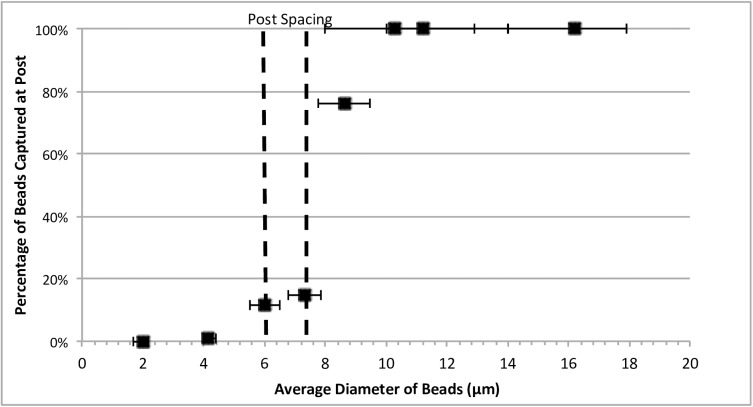
Determining percentage of beads captured between the post spacing with varying bead size. 100% trapping of the beads occurred with 10 μm diameter beads suggesting variance in bead size and deformation of posts affected trapping rate. Each bead size had a total of 150–200 beads to obtain data point of percentage of beads captured. Horizontal error bars are standard deviation from average bead diameter obtained from manufacturer.

To test the optimal bead range for capturing at the posts, beads ranging from 2 μm to 16 μm were introduced into the device’s filtering region and the number of beads captured was counted manually via microscopy. The different sized particles will determine the threshold at which all beads will be captured to ensure no beads are lost during the capture process. For device post spacing of 6 μm, beads of 6 and 7 μm diameters were captured at 10–20% efficiency (Fig 3). This low capture rate can be attributed to the fact that the constriction size is not precisely 6 μm. Measurement of gap size by image analysis shows the constriction size can increase up to 8 μm. During the fabrication process, the posts are distorted due to the elastic nature of PDMS. At bead sizes greater than 10 microns, 100% of the beads were captured at the posts. For maximum capturing efficiency, beads to be captured should be 50% greater than the designed gap size.

### Targeted particle binding to functionalized beads

**Fig 4:**
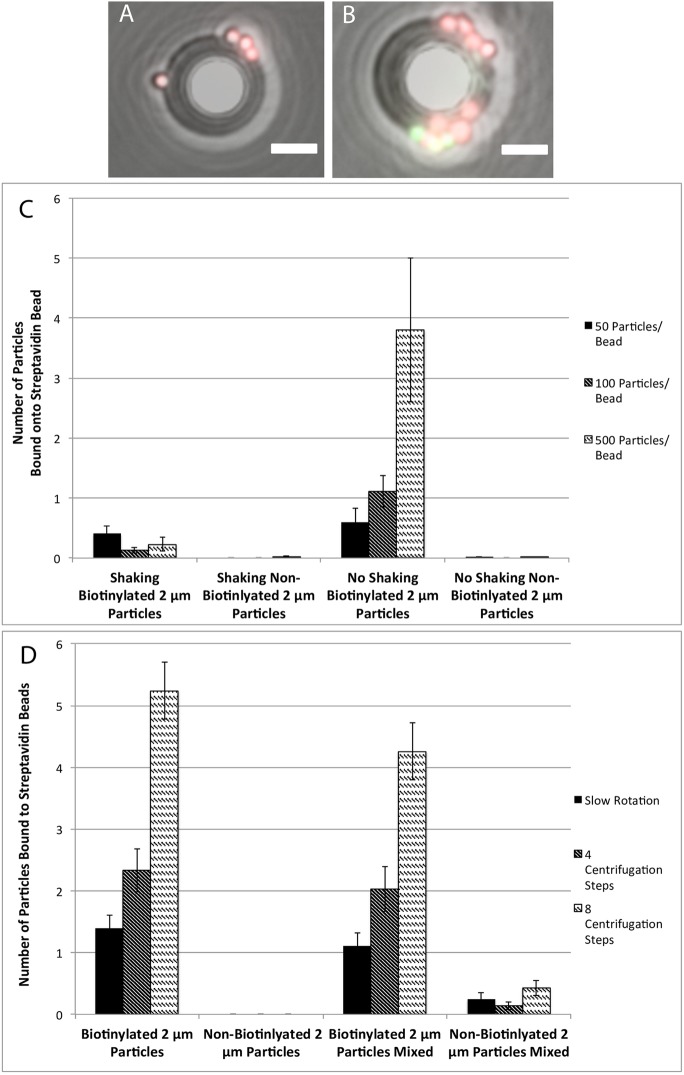
Validation of targeted particle binding at various conditions. (A-B) Images of targeted and non-targeted particles bound onto beads after filtration. Targeted particles fluoresce red and non-targeted particles fluoresce green. Scale bar is 8 μm. (C) Binding of particles to beads using streptavidin-biotin bond with shaking and no shaking conditions with varying ratios of particles to beads. Compared to a control with non-biotinylated particles. (D) Binding of particles to beads using different techniques to optimize binding. Trials included adding targeted and non-targeted particles together with beads to look at specificity. Trials performed at 100 particles per bead. All error bars are standard error.

Optimal conditions for binding biotinylated particles to streptavidin-functionalized beads were determined to ensure efficient separation of targets from non-targets in droplets. Polystyrene beads with streptavidin-functionalized surface were mixed with polystyrene particles with biotin-functionalized surface in the aqueous phase to determine the maximum extent to which they bind. Pink fluorescent biotinylated particles were used as the target and green fluorescent non-biotinylated particles as the non-target (Fig 4A and 4B).

There were several factors that influenced the binding of the target and non-target particles to the beads (Fig 4C). For particles successfully bound, increasing the ratio of biotinylated particles to streptavidin beads proportionally increased the number of particles bound. Also, on average, no shaking during binding improves the number of particles bound most likely due to decreased shear between the particles. In addition, the absence of shaking causes the beads and particles to precipitate to the bottom of the tube and allows for extended contact between them. Overall, the number of particles bound was observed to be lower than cells. This is likely due to the short lengths of the streptavidin and biotin molecules [32,33] and steric hindrance from the size of the particles [34,35]. Since the beads settle in layers, there is uneven exposure of the biotinylated particles to the streptavidin beads. This phenomenon accounts for the high variance of the number of particles bound to individual streptavidin beads.

Further optimization of binding by changing incubation parameters led to increased binding capacity and high specific binding (>90%). A series of experiments revealed conditions that would result in more particles bound while decreasing the variance (Fig 4D). In these experiments, both biotinylated and non-biotinylated fluorescent particles were used to determine the specificity. Using a rotator set at 60 rpm to increase contact time of particles to beads while still mixing the beads, the number of biotinylated particles bound increased by 33%, but the variance across beads remained high. Therefore, to increase contact between biotinylated particles and streptavidin beads while still maintaining uniform surface contact, beads were repeatedly centrifuged down into a pellet and re-suspended, binding increased five fold using this technique and variance halved. The four and eight centrifugation/re-suspension techniques offered higher specificity (95–96%) than rotating at 60 rpm (86%).

### Targeted particle separation

**Fig 5:**
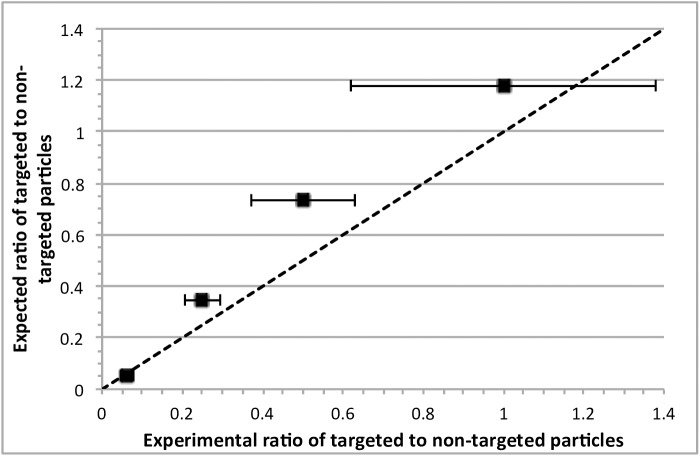
Distribution of targeted to non-targeted particles in droplets once generated. Dotted line indicates the calculated average ratio expected. Total number of beads in each droplet was set to 25. 22 droplets were used for each data point. All error bars are standard error.

Beads and particles encapsulated in surfactant-stabilized droplets follow the Poisson distribution. The distribution of targeted and non-targeted particles in each droplet is shown in Fig 5 for four conditions with a constant total number of beads and particles. Under each condition, varying ratios of targeted vs. non-targeted particles were observed across different droplets. However, the average value was close to the expected ratio, showing that the average number and ratio of particles and beads in each droplet can be manipulated effectively.

**Fig 6:**
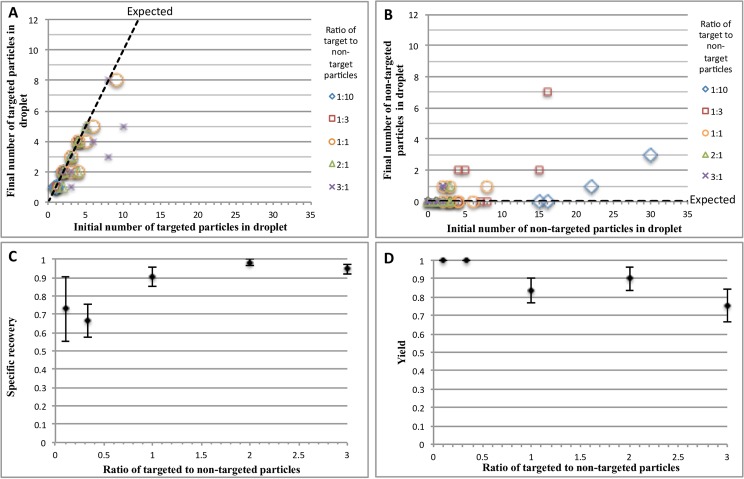
Separation of targeted particles from droplets and analysis of efficacy. (A) Number of the targeted particles bound onto the streptavidin beads. (B) Number of the non-targeted particles bound onto the streptavidin beads. All ratios shown are ratio of targeted to non-targeted particles in individual droplets. (C) Specificity of binding the target particles to the streptavidin beads. Specific recovery determined by final number of targeted particles bound compared to the total number of particles bound. (D) Yield of the number of targeted particles bound to streptavidin beads. Yield determined by the final number of targeted particles bound compared to initial number of targeted particles in the droplet. A total of ten droplets were separated for replicates. All error are standard error.

The device described in Fig 2A was tested with targeted and non-targeted particles to determine the separation efficiency. The streptavidin beads were able to bind the majority of targeted particles, as shown in Fig 6A. Experiments conducted to optimize particle-to-bead binding described in Fig 4B demonstrated that optimally, an average of 5-targeted particles could be bound onto a single streptavidin bead. This result was consistent with what was observed in droplets as increasing the number of initial targeted particles resulted in a rough maximum equivalent to the 5-particle limit. To increase the number of target particles captured, the number of beads per droplet can be increased. We have successfully used up to 10 beads per droplet to capture particles, and have re-encapsulated all of the beads. Overall, the system is able to specifically recover up to 98% of the targeted particles in droplets. Note that, for non-targeted particles, less than 10% of the particles bound to streptavidin beads (Fig 6B). Even with non-specific binding, it was shown that the re-encapsulation process was able to remove many of the non-specifically bound non-targeted particles. In principle, it is possible to remove essentially all the non-specifically bound non-targets by applying the washing procedure multiple times to a droplet.

To define the efficiency of the system, two metrics were used: specific recovery and specific yield.

$$Specific\;Yield=\frac{Number\;of\;Targeted\;Microparticles\;Bound\;After\;Separation}{Number\;of\;Targeted\;Microparticles\;in\;Droplet\;Before\;Separation}$$ (2)

$$Specific\;Recovery=\frac{Number\;of\;Targeted\;Microparticles\;Bound\;After\;Separation}{Number\;of\;Total\;Microparticles\;Bound\;After\;Separation}$$ (3)

As shown in Fig 6C, the specific recovery of targeted particles approached 100% for higher ratios of targeted to non-targeted particles. However, the yield decreased at these higher ratios (Fig 6D). This is an expected trend, as higher number targeted particles will result in saturation of the binding sites on the beads, and the total number of beads per particle can be adjusted depending on the application. Also, the lower specificity at lower target to non-target ratios can be improved using multiple separation and recovery steps.

## Conclusion

This device is a pneumatic droplet washing system capable of separating targets from non-targets in a droplet. We determined optimum parameters for the device and beads and demonstrate successful binding of the target both on and off-chip. We demonstrated successful operation of the device and characterized the separation efficiency. Using streptavidin surface-functionalized particles, we were able to capture up to 100% of the biotinylated particles under certain conditions. The operation requires no external magnetic or electrical fields for performing the washing process. This reduces the cost of fabrication and operation of the device by only requiring pneumatic lines. Without the need for magnetic particles, a wide variety of bead materials and surface-chemistry can be used increasing the versatility. We have shown proof-of-concept using particles, but many applications are possible. The bead-based system allows for versatility of the surface chemistry and can be applied to separate particles in surfactant-stabilized droplets. In addition, it is possible to use different surface-chemistry beads in the same droplet, allowing for the multiplexing of assays in a single droplet. Operating the device at higher flow rates and adding an additional channel for removing separated droplets can increase throughput. The device can also be automated in the future for faster throughput washing by using measuring viscosity difference between the oil and aqueous phase to automate the operation.

## Supporting information

S1 Fig Plot of Fig 6A and 6B separated into individual plots. Ratios are given in targeted particles to non-targeted particles. Figs A-E are targeted particles bound. Expected number of particles is shown by a dotted line on the graph. (A) 1:10 (B) 1:3 (C) 1:1 (D) 2:1 (E) 3:1 Figs F-J are non-targeted particles bound. (F) 1:10 (G) 1:3 (H) 1:1 (I) 2:1 (J) 3:1. Separation on the device was performed for a total of ten droplets for each ratio.
(TIF)

S2 Fig Image of a droplet after separation showing two captured biotinylated particles (in pink) in the new droplet. Scale bar is 100 μm.
(TIF)

S1 File Bead capture at device capture region. (M4V)

S2 File Bead re-encapsulation at device capture region. (M4V)
