# Supplementary figures and images for: Bead mediated separation of microparticles in droplets
Source: PLoS One. 2017 Mar 10;12(3):e0173479. doi: 10.1371/journal.pone.0173479 (PMC5345812; doi:10.1371/journal.pone.0173479)

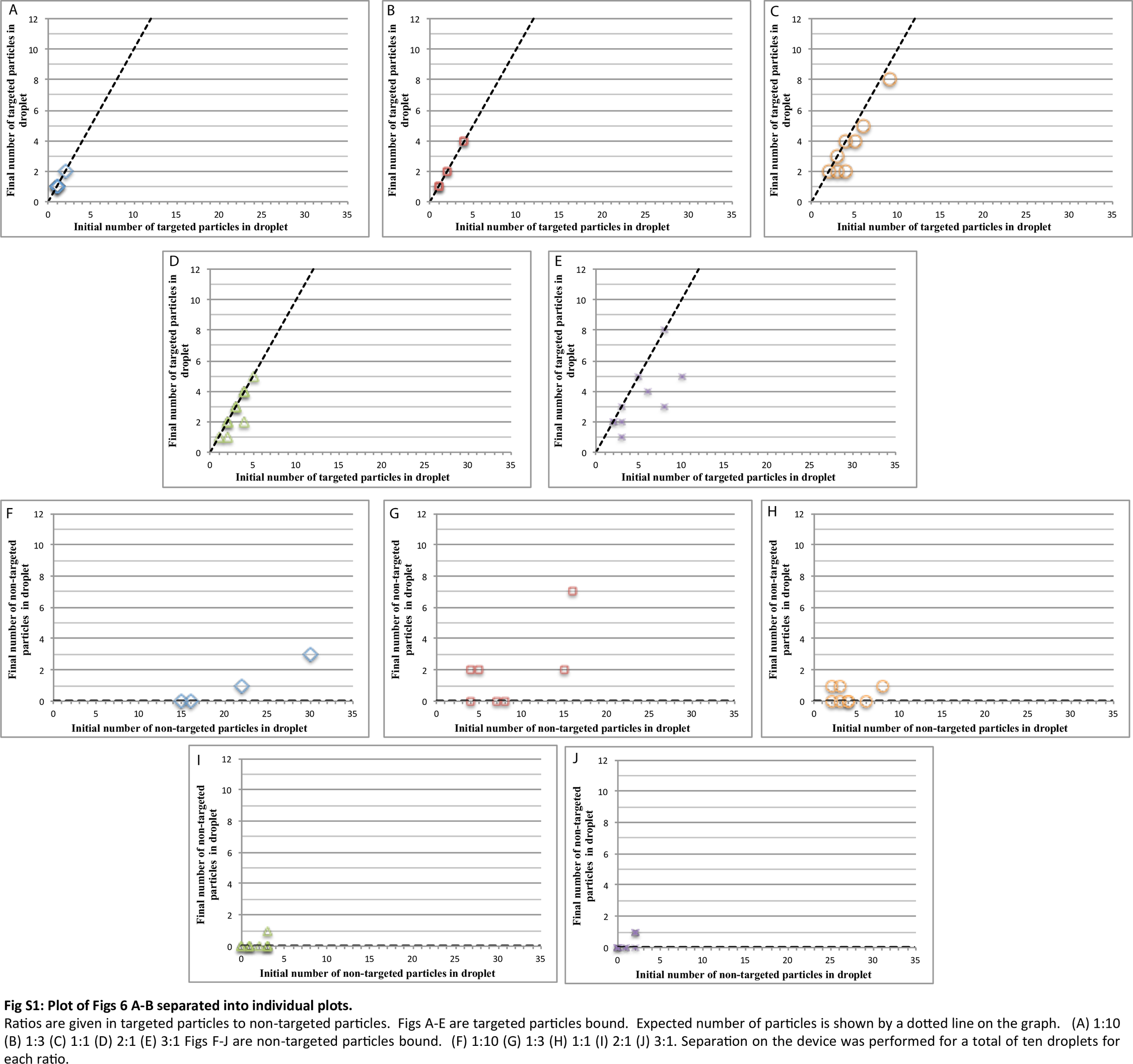

Supplement: S1 Fig — Ratios are given in targeted particles to non-targeted particles. Figs A-E are targeted particles bound. Expected number of particles is shown by a dotted line on the graph. (A) 1:10 (B) 1:3 (C) 1:1 (D) 2:1 (E) 3:1 Figs F-J are non-targeted particles bound. (F) 1:10 (G) 1:3 (H) 1:1 (I) 2:1 (J) 3:1. Separation on the device was performed for a total of ten droplets for each ratio. (TIF) [file pone.0173479.s001.tif]

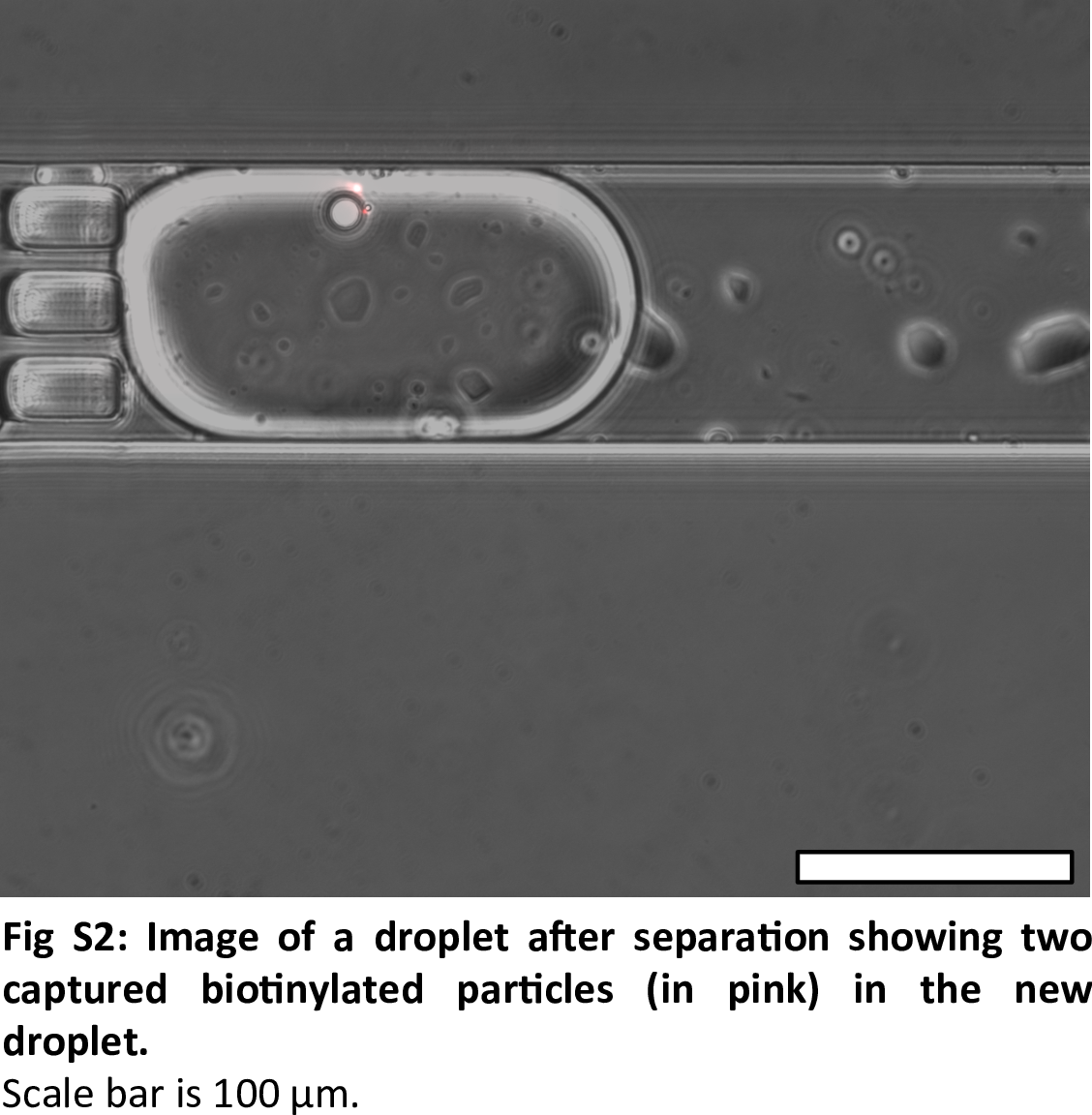

Supplement: S2 Fig — Scale bar is 100 μm. (TIF) [file pone.0173479.s002.tif]
